# Pyroptosis endotypes and nonlinear biomarker-mortality relationships in older adults with community-acquired pneumonia: the amplifying role of malnutrition

**DOI:** 10.3389/fimmu.2026.1770810

**Published:** 2026-04-14

**Authors:** Jingxian Liao, Chenghu Ning, Yinglan Zhu, Chen Gong, Chunhui Xie, Lei Miao

**Affiliations:** 1 Department of Geriatrics, The Second People's Hospital of Lianyungang Affiliated to Kangda College of Nanjing Medical University, Lianyungang, China; 2 Department of Radiology, The Second People's Hospital of Lianyungang, Lianyungang, China; 3 Nanjing Hospital of Traditional Chinese Medicine, Nanjing Hospital of Chinese Medicine Affiliated to Nanjing University of Chinese Medicine, Nanjing, China; 4 Department of Critical Care Medicine, The Second People's Hospital of Lianyungang, Lianyungang, China

**Keywords:** community-acquired pneumonia, gasdermin D, inflammasome activation, NLRP3 inflammasome, nutritional immunology, pyroptosis

## Abstract

**Background:**

Despite their disproportionately high mortality from community-acquired pneumonia (CAP), older adults remain understudied regarding inflammasome-mediated cell death pathways. We sought to determine whether distinct pyroptosis activation patterns exist in this population and how nutritional status modifies their prognostic impact.

**Methods:**

This retrospective cohort study enrolled 282 patients aged ≥75 years hospitalized for CAP. We quantified circulating pyroptosis effectors (gasdermin D [GSDMD], NLRP3, caspase-1) and assessed nutritional status using the Mini Nutritional Assessment-Short Form (MNA-SF). K-means clustering identified biological endotypes; generalized additive models (GAMs) characterized nonlinear biomarker-mortality relationships. The primary endpoint was 28-day all-cause mortality.

**Results:**

Three pyroptosis endotypes emerged with markedly divergent outcomes: hyper-pyroptotic (n=73; mortality 57.5%), intermediate-pyroptotic (n=128; mortality 10.2%), and hypo-pyroptotic (n=81; mortality 1.2%). The hyper-pyroptotic endotype was characterized by severe malnutrition (48.2% with MNA-SF ≤7) and elevated cytokines (median IL-6: 98.4 pg/mL). GAM analysis revealed threshold-dependent, nonlinear relationships—mortality risk escalated sharply when GSDMD exceeded 3.5 ng/mL but showed attenuation at extreme values. Notably, two-dimensional analyses demonstrated supra-additive risk in patients with concurrent nutritional compromise and pyroptosis activation. An integrated prognostic model achieved AUC 0.898 (95% CI: 0.847–0.943), significantly outperforming the Pneumonia Severity Index alone (AUC 0.793; P<0.001), with superior net benefit across clinically relevant decision thresholds (10–30%).

**Conclusion:**

Geriatric CAP comprises biologically distinct pyroptosis endotypes. Malnutrition was associated with a stronger relationship between pyroptosis markers and short-term mortality, consistent with effect modification. These findings support integrating nutritional assessment with pyroptosis biomarker profiling for risk stratification and generate hypotheses for prospective mechanistic and interventional studies.

## Introduction

The aging immune system is characterized by a fundamental immunological paradox that has profound clinical consequences. On one hand, older adults exhibit chronic, low-grade systemic inflammation—a state termed “inflammaging”—driven by accumulation of senescent cells, altered gut microbiota composition, and persistent cytomegalovirus reactivation (1). On the other hand, pathogen-specific immune responses become increasingly blunted, reflecting thymic involution, exhausted T cell repertoires, and impaired innate immune cell function (2). This dual vulnerability creates a precarious immunological landscape: when confronted with acute infection, the aged immune system may preferentially mount exaggerated, nonspecific inflammatory responses rather than coordinated, pathogen-clearing immunity. Nowhere is this paradox more clinically evident than in community-acquired pneumonia (CAP), where older adults experience case fatality rates of 10–30%—five to ten-fold higher than younger populations—despite comparable pathogen virulence profiles (3, 4). Crucially, this mortality disparity cannot be attributed solely to comorbidity accumulation; rather, it reflects fundamental alterations in the architecture and regulation of innate immune responses, particularly in the calibration of inflammatory cell death pathways (5, 6). Understanding how aging reshapes the balance between protective antimicrobial immunity and destructive hyperinflammation has emerged as a central question in geriatric immunology—one with direct implications for risk stratification and host-directed therapeutic strategies.

Pyroptosis—an inflammatory form of programmed cell death—is executed through gasdermin D (GSDMD) pore formation, which releases interleukin-1β and IL-18 while inducing cell lysis (7, 8). This pathway is initiated by inflammasome complexes—supramolecular organizing centers in which pattern recognition receptors such as NLRP3 recruit and activate caspase-1 in response to pathogen-associated or danger-associated molecular patterns (9). In the context of pulmonary infection, pyroptosis serves an evolutionarily conserved protective function: by eliminating infected cells and releasing inflammatory cytokines, it recruits neutrophils and activates adaptive immunity to contain bacterial proliferation (10, 11). However, the relationship between pyroptosis intensity and clinical outcomes is not linear: excessive activation amplifies inflammatory cascades and disrupts alveolar barrier integrity, paradoxically impairing pathogen clearance (12, 13). This “pyroptosis paradox”—wherein both insufficient and excessive activation may prove detrimental—has been demonstrated in sepsis models (14) and suggested in COVID-19 acute respiratory distress syndrome (15), but remains poorly characterized in bacterial CAP, particularly among older adults whose baseline inflammasome regulation may already be perturbed.

The intersection of immunosenescence and pyroptosis regulation represents a critical yet understudied dimension of geriatric infectious disease immunology. Aging is associated with a paradoxical immune phenotype termed “inflammaging”—characterized by elevated basal inflammatory tone despite diminished pathogen-specific responses (16, 17). At the molecular level, aged macrophages exhibit heightened NLRP3 inflammasome priming, lower activation thresholds, and impaired resolution mechanisms, collectively predisposing to exaggerated pyroptotic responses upon infectious challenge (18, 19). Concurrently, immunosenescent lymphocytes provide suboptimal support for inflammasome containment, as regulatory T cell function and IL-10 production decline with age (20).

These age-related alterations suggest that the “pyroptosis thermostat” may be miscalibrated in older adults, potentially explaining clinical observations of heterogeneous outcomes in geriatric CAP. Some patients mount appropriately calibrated inflammatory responses and recover, while others—ostensibly similar in clinical presentation—spiral into hyperinflammation and multiorgan dysfunction. Identifying biomarkers that distinguish these trajectories could transform prognostication from reactive pattern recognition to mechanistically informed risk stratification.

Nutritional status represents an underappreciated modifier of inflammasome regulation in geriatric pneumonia. Malnutrition—affecting 30–60% of hospitalized older adults—actively shapes immune function through multiple converging pathways (21, 22). Protein-energy malnutrition impairs autophagy, the cellular process that normally constrains inflammasome activation by clearing damaged mitochondria (23, 24). Micronutrient deficiencies compound this effect: zinc deficiency disinhibits NLRP3 assembly (25), while vitamin D insufficiency removes tonic suppression of inflammasome priming (26, 27). These observations suggest a mechanistic framework in which malnutrition may lower the threshold for pathological pyroptosis activation—converting a protective antimicrobial response into tissue-destructive inflammation. If correct, this predicts that pyroptosis biomarker prognostic significance should vary according to nutritional status—a nonlinear interaction potentially obscured by conventional analytical approaches.

Frailty and malnutrition are increasingly recognized as key determinants of prognosis in older adults hospitalized with community-acquired pneumonia, beyond conventional severity scores (28). However, how nutritional vulnerability relates to inflammasome/pyroptosis-linked host injury patterns in humans remains incompletely characterized. Against this background, we designed this study with dual objectives. First, we aimed to test whether older adults with CAP encompass biologically distinct pyroptosis endotypes with divergent outcomes, rather than representing a homogeneous population with uniformly elevated inflammation. Second, we hypothesized that biomarker-mortality relationships follow nonlinear trajectories and that nutritional compromise amplifies pyroptosis-associated risk through synergistic interactions. By addressing these questions, we sought to develop a clinically applicable risk stratification framework that integrates inflammasome biology with nutritional assessment.

## Methods

### Study design and patients

We conducted a single-center, retrospective cohort study at the Second People’s Hospital of Lianyungang, enrolling consecutive adults aged 75 years or older who were hospitalized with community-acquired pneumonia (CAP) between 1 January 2020 and 1 January 2025. CAP was defined according to American Thoracic Society/Infectious Diseases Society of America (ATS/IDSA) criteria as a new pulmonary infiltrate on chest imaging together with at least one compatible clinical feature (cough, sputum production, fever, dyspnea, or abnormal auscultation) (29). Patients were eligible if they met the age and CAP criteria and had baseline nutritional assessment and core laboratory data available from the index admission.

Notably, this single-center study was conducted in a Chinese tertiary hospital serving the Lianyungang metropolitan area; the patient population was predominantly of Han Chinese ethnicity. This demographic context should be considered when interpreting biomarker distributions and their generalizability, as discussed in the Limitations section.

We excluded patients with conditions likely to distort host inflammatory responses or confound biomarker interpretation: hospital-acquired pneumonia; established immunosuppression (e.g., HIV infection, primary or secondary immunodeficiency) or current/recent immunosuppressive therapy within 3 months (systemic corticosteroids, cytotoxic chemotherapy, or biologic agents); hematologic malignancy, solid-organ transplantation, or active solid tumors receiving antineoplastic therapy; and active autoimmune or connective tissue diseases requiring immunosuppression or in clinical flare. Additional exclusions were decompensated end-organ disease (New York Heart Association class IV heart failure, end-stage renal disease on dialysis, Child–Pugh class C cirrhosis, unstable coronary syndromes, or acute stroke within 3 months); recent major surgery, trauma, or blood transfusion within 30 days; and incomplete essential clinical or outcome information.

Case ascertainment used electronic medical record queries of diagnostic codes and radiology reports, followed by manual chart review by trained investigators. For patients with multiple admissions during the study window, only the first eligible (index) hospitalization was included. Baseline clinical variables, nutritional assessments, and blood sampling were obtained as part of routine care within 24 hours of admission. All clinical variables, nutritional assessments, and biomarker measurements analyzed in this study were obtained at this single baseline time point; serial or longitudinal biomarker sampling was not performed. Consequently, the analytic framework is cross-sectional with respect to biomarker and nutritional assessment, and all associations reported herein reflect baseline status rather than temporal trajectories.

The study complied with the Declaration of Helsinki and was approved by the Institutional Review Board of the Second People’s Hospital of Lianyungang (Approval No. 2024K020); the requirement for informed consent was waived owing to the use of anonymized, pre-existing clinical data.

### Data collection and definitions

Two trained investigators independently abstracted data from the electronic medical record (EMR) using a piloted case report form and standard operating procedures. Discrepancies were resolved by consensus, with arbitration by a senior investigator when necessary.

Baseline variables included demographics (age, sex) and clinical characteristics obtained at admission. Body mass index (BMI, kg/m²) was calculated from bedside height and weight; when bedside measurement was not feasible, the most recent measurement within 30 days prior to admission was used. Comorbidities (hypertension, diabetes, chronic obstructive pulmonary disease, chronic kidney disease, and cerebrovascular disease) were identified from physician-documented diagnoses and corroborated by ICD-10 codes. The Pneumonia Severity Index (PSI) was computed at admission according to the original specification.

Microbiological evaluation followed institutional protocols. When feasible, sputum and two sets of peripheral blood cultures were obtained prior to antimicrobial therapy. Sputum quality was assessed by light microscopy, and suboptimal samples were flagged. Additional tests—including urine antigens for Streptococcus pneumoniae and Legionella pneumophila and respiratory viral PCR—were ordered at the discretion of the treating clinicians. Etiology was classified as bacterial, viral, mixed, or undetermined based on positive culture, antigen, or PCR results from appropriate specimens. Likely contaminants (for example, a single coagulase-negative staphylococcal blood culture without corroborating evidence) were treated as negative.

Nutritional status was assessed within 24 hours of admission using the Mini Nutritional Assessment–Short Form (MNA-SF; range 0–14) (30). MNA-SF was analyzed both as a continuous score and by prespecified categories: normal (12–14), at risk of malnutrition (8–11), and malnutrition (0–7). Serum albumin was measured by the hospital’s accredited clinical laboratory on an automated chemistry analyzer as an objective nutritional biomarker.

The primary outcome was 28-day all-cause mortality from the date of hospital admission. Vital status was ascertained from electronic records and, when needed, confirmed by telephone follow-up with patients or surrogates; deaths occurring after interfacility transfer were verified with receiving institutions. Patients without documented death were censored at 28 days or last contact, whichever occurred first. All data were deidentified prior to analysis. Patterns of missingness were profiled and addressed as prespecified in the Statistical Analysis section.

### Biomarker selection and enzyme-linked immunosorbent assay

We assembled a biomarker panel targeting three levels of the pyroptosis cascade to capture pathway activation comprehensively:

Inflammasome sensor level: NLRP3 protein, the most extensively characterized inflammasome sensor in pulmonary infection, was selected given its established role in bacterial recognition and sterile inflammation amplification.

Effector caspase level: Caspase-1, the canonical inflammatory caspase downstream of NLRP3, was quantified to assess inflammasome enzymatic activation status.

Executioner level: Gasdermin D, the pore-forming effector protein whose cleavage represents the committed step in pyroptotic cell death, was measured as the most proximal marker of pyroptosis execution.

Downstream cytokine level: IL-1β and IL-18, the signature cytokines released through GSDMD pores, were quantified to assess the functional inflammatory output of pyroptosis activation.

This multi-level approach was designed to distinguish scenarios of inflammasome priming without activation (elevated NLRP3 alone) from complete pathway engagement (concordant elevation across all markers).

Venous blood samples were collected within 24 hours of hospital arrival as part of the routine admission workup. Blood was drawn before the first antibiotic dose in 78.4% of patients (221/282); in the remaining 61 patients, empirical antibiotics had already been started in the emergency department before admission blood work was completed. The retrospective design precluded capture of a precise timestamp linking emergency department registration to phlebotomy, though institutional practice was to complete admission blood draws within the first few hours of arrival. Pre-admission symptom duration was not recorded in a structured EMR field and could not be reliably extracted from free-text notes. Blood was drawn into EDTA-anticoagulated tubes, centrifuged at 1500×g for 15 minutes at 4 °C within 2 hours of collection, and plasma aliquots were stored at −80 °C until batch analysis. Biomarker quantification was based on this single admission sample; serial sampling was not feasible given the retrospective design and routine clinical workflow.

Pyroptosis-related proteins were quantified using commercially available sandwich ELISA kits: Human GSDMD ELISA Kit (detection range: 0.156–10 ng/mL; intra-assay CV <8%, inter-assay CV <10%); Human NLRP3 ELISA Kit (detection range: 0.625–40 ng/mL; intra-assay CV <8%, inter-assay CV <10%); Human Caspase-1 ELISA Kit (detection range: 3.2–200 pg/mL; intra-assay CV <8%, inter-assay CV <10%). The GSDMD ELISA kit employed in this study detects total circulating GSDMD protein, encompassing both the full-length precursor (53 kDa) and the caspase-1-cleaved N-terminal fragment (GSDMD-NT, 31 kDa), as the antibodies target epitopes within the N-terminal domain. This assay does not discriminate between inactive and active forms. However, the strong positive correlation between circulating GSDMD levels and caspase-1, IL-1β, and IL-18—all indicators of active inflammasome engagement—suggests that elevated total GSDMD levels in our cohort predominantly reflect caspase-1-mediated cleavage and active pyroptosis rather than passive release of uncleaved precursor.

Inflammatory cytokines IL-1β and IL-18 were quantified using ELISA kits from the same manufacturer. IL-6 was measured using the hospital’s routine clinical chemistry platform.

All samples were assayed in duplicate according to manufacturer protocols. Samples with coefficient of variation >15% between duplicates were re-assayed. Values below the lower limit of detection (LLOD) were assigned LLOD/√2 for statistical analysis, following FDA bioanalytical method validation guidelines. No samples exceeded the upper limit of detection. Quality control samples at low, medium, and high concentrations (provided by manufacturers) were included on each plate, with all QC values falling within ±2 standard deviations of target values.

IL-1β, IL-18, and IL-6 were deliberately excluded from the clustering algorithm because they are pleiotropic cytokines produced through multiple inflammatory pathways beyond the NLRP3–caspase-1–GSDMD axis; their inclusion would risk conflating pyroptosis-specific heterogeneity with broader inflammatory variation.

### Sample size considerations

This was a retrospective cohort study, and sample size was determined by the number of eligible patients during the study period. *Post hoc* power analysis confirmed adequate statistical power: with 282 patients and 56 events (19.9% mortality), we had >80% power to detect an AUC of ≥0.75 for the primary predictive model (two-sided α=0.05). For clustering analysis, our smallest cluster (n=73) exceeded the recommended minimum of 50 observations per cluster for stable three-cluster solutions.

### Statistical analysis

Continuous variables were assessed for distribution (Shapiro–Wilk tests, histograms, Q–Q plots) and summarized as mean ± SD (approximately normal) or median [IQR] (skewed). Categorical variables were counts (%). Between-group comparisons used Student’s t-test or Mann–Whitney U test for continuous variables and χ² or Fisher’s exact test for categorical variables. Spearman correlations evaluated associations among clinical, nutritional, and biomarker variables. Prior to modeling, continuous predictors were standardized (z-scores). Multicollinearity was screened using variance inflation factors and pairwise correlations.

Unsupervised K-means clustering was applied to identify pyroptosis-related phenotypic subgroups among older adults with CAP. The three core pyroptosis-executing biomarkers (GSDMD, NLRP3, CASP1) were first standardized via z-score transformation to ensure equal weighting, and K-means clustering was then performed using the Hartigan–Wong algorithm with 25 random starts and a maximum of 100 iterations. The optimal number of clusters was determined by inspecting the within-cluster sum of squares (WSS) across k = 2–10 (elbow method); the inflection point—where marginal WSS reduction began to plateau—guided the initial selection. This choice was independently corroborated by the gap statistic, computed via the clusGap function (cluster package, B = 500 reference datasets) for k = 1–10.

Cluster assignment stability was assessed through bootstrap resampling: 1,000 bootstrap samples (n = 282, drawn with replacement) were generated, and the full clustering pipeline (z-score standardization followed by K-means with the selected k and 25 random starts) was repeated for each sample. The adjusted Rand index (ARI) between bootstrap-derived and original assignments was calculated per iteration; the distribution of ARI values served as an empirical measure of reproducibility. The average silhouette width for the final solution was computed to evaluate within-cluster cohesion relative to between-cluster separation, and principal component analysis (PCA) was used to project the biomarker space into two dimensions for visual inspection of cluster distinctness, with 95% confidence ellipses overlaid. Final cluster assignments were appended to the original dataset for all subsequent analyses. All clustering procedures were carried out in R (version 4.5.1) using the factoextra, cluster, and fmsb packages.

To examine independent nonlinear relationships with 28-day mortality, we fitted GAMs with a logit link: logit(Pr[mortality]) ~ s(MNA-SF, k=4) + s(NLRP3, k=4) + s(GSDMD, k=4) + s(CASP1, k=4) + Age + Sex, where s(.) denotes thin−plate regression splines limited to four basis functions (31). We focused on main−effect smooths to avoid overparameterization and instead explored potential joint patterns by plotting two−dimensional partial−dependence surfaces. The basis dimension (k=4) was selected *a priori* to permit detection of modest nonlinearity (U-shaped or threshold patterns) while guarding against overfitting.

For prognostic modeling, we used LASSO logistic regression (glmnet) with ten-fold cross-validation to select predictors, followed by a multivariable logistic regression including selected variables (32). The penalty parameter λ was selected using the one-standard-error rule (λ.1se criterion) from 10-fold cross-validation, which identifies the most parsimonious model within one standard error of the minimum cross-validated deviance—a strategy that balances discrimination with model simplicity and reduces overfitting risk. Discrimination was assessed by area under the ROC curve (AUC; pROC), and calibration by calibration plots and the Hosmer–Lemeshow goodness-of-fit test.

Decision-curve analysis (DCA) was used to assess the clinical utility of the final multivariable model by quantifying the net benefit across a range of threshold probabilities for 28−day mortality, and comparing it with default strategies (treat all, treat none) and the PSI alone (33). DCA was implemented in R using the rmda package, with threshold probabilities pre-specified based on plausible clinical decision thresholds in older adults with CAP.

All analyses were carried out in R statistical software (version 4.5.1), utilizing the ‘mgcv’ package for GAM, ‘glmnet’ for LASSO regression, and ‘pROC’ for ROC analysis. A two-sided p-value <0.05 was considered statistically significant.

### Handling missing data

All datasets were systematically reviewed for completeness and internal consistency prior to analysis. Variables with more than 20% missingness across the cohort were excluded *a priori* to limit the risk of bias introduced by excessive imputation uncertainty. The primary outcome (28-day all-cause mortality) was not imputed. Patterns and mechanisms of missingness were explored via descriptive summaries and visualization; analyses proceeded under a missing at random (MAR) assumption.

For variables retained in the analysis with incomplete observations, multiple imputation by chained equations (MICE) was performed using the mice package in R (version 4.5.1). Five imputed datasets (m = 5) were generated with 50 iterations to promote algorithmic convergence and preserve between-imputation variability. The imputation model included all variables later used in statistical analyses as predictors (age, sex, BMI, PSI, MNA-SF, albumin, IL-6, IL-1β, IL-18, GSDMD, NLRP3, CASP1, comorbidities, and etiology), as well as the outcome indicator as a predictor but not as a target for imputation, to improve plausibility under MAR.

Imputation models were specified by data type. Continuous variables (e.g., albumin, IL-6, IL-1β, IL-18, GSDMD, NLRP3, CASP1, BMI, PSI) were imputed using predictive mean matching (PMM). Binary categorical variables (e.g., sex, presence of comorbidities) were imputed via logistic regression, and nominal multi-category variables (e.g., etiological classification: bacterial, viral, mixed, undetermined) via polytomous logistic regression. The MNA-SF score (0–14) was treated as a continuous variable and imputed using PMM; after imputation, values were truncated to the valid range and, when required for descriptive summaries, categorized into prespecified groups (malnutrition 0–7, at risk 8–11, well-nourished 12–14).

All subsequent statistical procedures were conducted separately within each imputed dataset, including between-group comparisons, Spearman correlations, generalized additive models (GAMs), least absolute shrinkage and selection operator (LASSO) variable selection, and multivariable logistic regression. Continuous predictors were standardized to z-scores after imputation and prior to modeling. Parameter estimates and standard errors were combined across imputations using Rubin’s rules to obtain pooled effect estimates and inferences. Model performance metrics (e.g., AUC and calibration) were summarized across imputed datasets using standard pooling procedures. Notably, all biomarkers and clinical variables in this study were measured at baseline; therefore, longitudinal or time-series–specific imputation was not required.

## Results

### Identification of pyroptosis-related subgroups and cluster characterization

**Figure 1:**
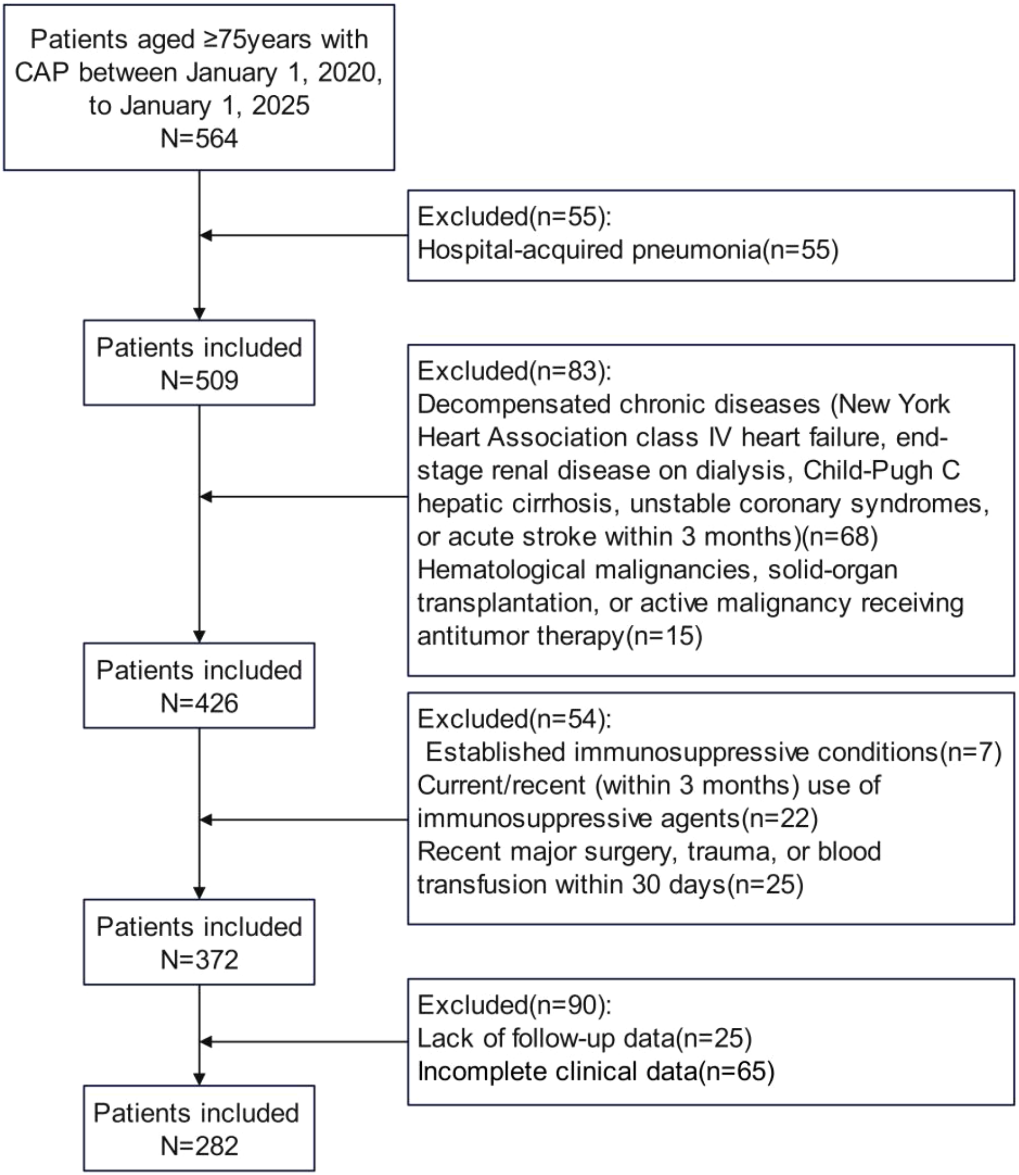
Flow diagram displaying the progress of all participants through the study.

**Figure 2:**
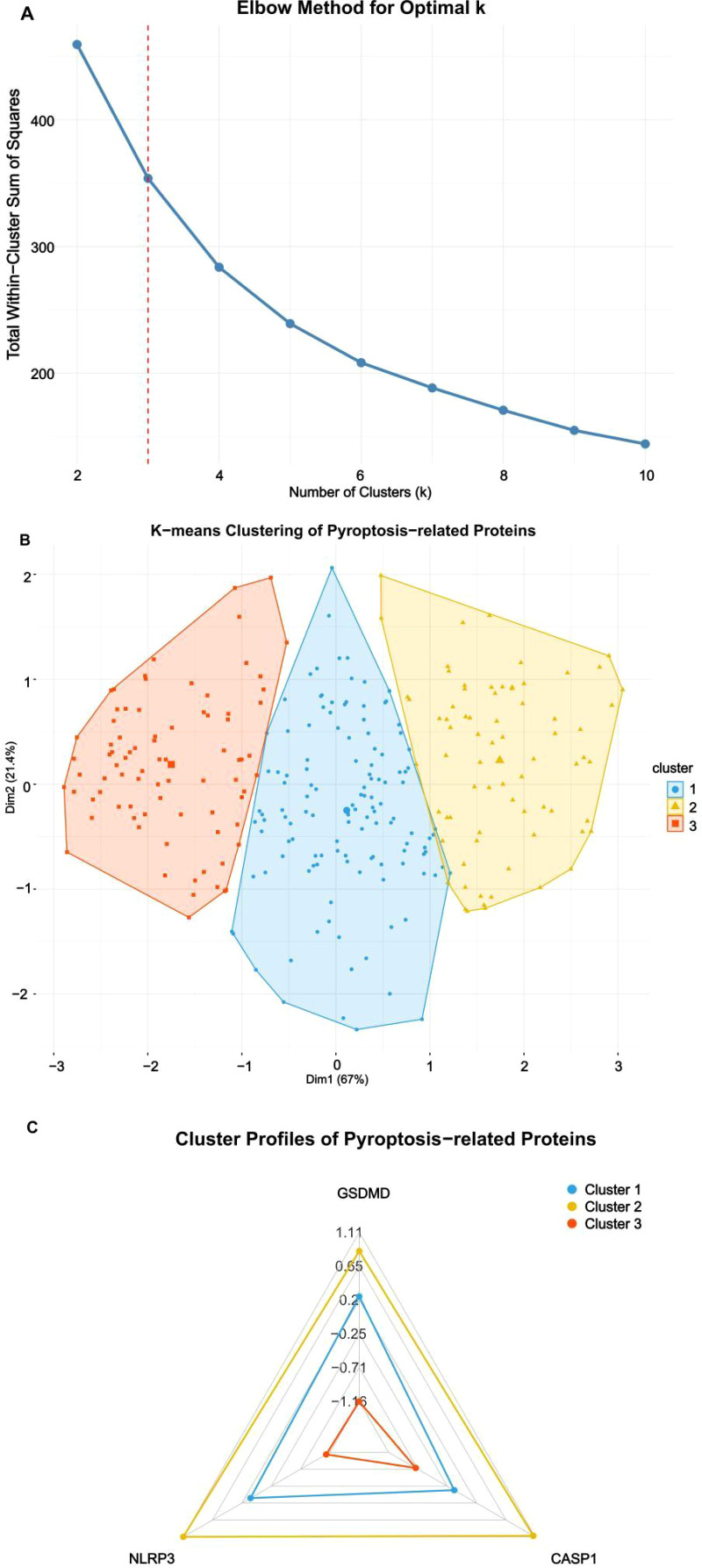
Identification and characterization of pyroptosis phenotypes in older adults with CAP. **(A)** Elbow method analysis determining the optimal cluster number. The inflection point at k=3 (red dashed line) indicates the point of diminishing returns in within-cluster sum of squares reduction, establishing three as the optimal number of pyroptosis-related phenotypes. **(B)** Principal Component Analysis (PCA) visualization of the three pyroptosis phenotypes. Each point represents an individual patient (n=282), with ellipses denoting 95% confidence intervals. Clear spatial separation along principal components 1 and 2 (accounting for 88.4% total variance) demonstrates distinct biological clustering. **(C)** Radar plot analysis of standardized protein expression profiles. Each axis represents one pyroptosis-related protein, with values indicating z-score transformed expression levels. Data points represent cluster centroids ± SEM. Each axis represents one pyroptosis-related protein, with values displayed as standardized z-scores (z = [x − μ]/σ; dimensionless units).

A total of 282 older adults with CAP met eligibility criteria and were included in the analysis (**Figure 1**). Unsupervised clustering analysis of three central pyroptosis-executing proteins (GSDMD, NLRP3, and CASP1) revealed three clinically relevant endotypes among older adults with CAP, demonstrating significant heterogeneity in pyroptosis activation patterns. The elbow method demonstrated an optimal k value of 3, as evidenced by the inflection point where the reduction in within-cluster sum of squares began to plateau (**Figure 2A**), indicating optimal cluster cohesion and separation. To assess cluster stability, we performed bootstrap resampling (1000 iterations) with adjusted Rand index calculation, which demonstrated high reproducibility (mean ARI = 0.78, 95% CI: 0.71–0.84). The three-cluster solution was further validated using the gap statistic, which confirmed k=3 as optimal. This selection was further verified by the clear separation of clusters observed in the principal component analysis (PCA) plot. PCA revealed clear separation among the three clusters in two-dimensional space (**Figure 2B**), accounting for 88.4% of total variance (PC1: 67%, PC2: 21.4%).

The three endotypes exhibited distinct biological signatures with clear clinical implications (**Table 1**; **Figure 2C**). The Hyper-pyroptotic phenotype (Cluster 2; n=73, 25.9%) was characterized by concordantly elevated expression of all three pyroptosis markers (mean z-scores: GSDMD = 3.97, NLRP3 = 3.94, CASP1 = 1.86), suggesting robust, coordinated activation of the canonical inflammasome-pyroptosis cascade. The Intermediate-pyroptotic phenotype (Cluster 1; n=128, 45.4%) displayed moderate marker elevations (GSDMD = 3.36, NLRP3 = 2.93, CASP1 = 1.16), potentially representing a transitional state or partial pathway engagement. The Hypo-pyroptotic phenotype (Cluster 3; n=81, 28.7%) exhibited uniformly low marker expression (GSDMD = 1.95, NLRP3 = 1.80, CASP1 = 0.82), consistent with attenuated pyroptotic activity—whether reflecting a milder infectious insult, preserved regulatory mechanisms, or immunosenescent hyporesponsiveness. PCA further confirmed the clear spatial separation of the three phenotypes in multidimensional space (**Figure 2B**). ANOVA tests indicated that mean expressions of all proteins (GSDMD, NLRP3, CASP1) were significantly different across clusters (p < 0.001).

**Table 1:** Cluster characterization and nomenclature.

Cluster	Phenotype	n	GSDMD z-score (SD)	NLRP3 z-score (SD)	CASP1 z-score (SD)
Cluster 1	Intermediate-pyroptotic	128	3.36 (0.67)	2.93 (0.60)	1.16 (0.43)
Cluster 2	Hyper-pyroptotic	73	3.97 (0.63)	3.94 (0.56)	1.86 (0.41)
Cluster 3	Hypo-pyroptotic	81	1.95 (0.57)	1.80 (0.50)	0.82 (0.40)
*P*			< 0.001	< 0.001	< 0.001

Continuous variables for pyroptosis-related proteins GSDMD, NLRP3, and CASP1 are presented for the entire cohort as mean (standard deviation) of standardized z-scores, calculated using z = (x − μ)/σ. As a result, values are dimensionless and directly comparable across biomarkers; higher z-scores indicate higher circulating levels of the corresponding protein. P values refer to overall differences across the three groups.

GSDMD, gasdermin D; NLRP3, NOD-like receptor protein 3; CASP1, caspase-1; SD, standard deviation.

The biological profiles of these three endotypes invite mechanistic interpretation. The hyper-pyroptotic phenotype, with concordantly elevated GSDMD, NLRP3, and caspase-1, likely represents a state of maximal, coordinate inflammasome engagement—potentially reflecting overwhelming pathogen burden, loss of regulatory checkpoints, or both. The striking co-occurrence of severe malnutrition in this group (48.2% with MNA-SF ≤7 versus 1.9% in the hypo-pyroptotic group) suggests that nutritional compromise may contribute to this dysregulated phenotype, either by impairing autophagy-mediated inflammasome resolution or by lowering activation thresholds through micronutrient deficiencies. The intermediate endotype may represent a transitional state—patients captured mid-trajectory between controlled and dysregulated responses—or alternatively, a distinct biological subgroup with partial pathway engagement. The hypo-pyroptotic phenotype, characterized by attenuated biomarker expression and near-universal survival (98.8%), likely comprises patients with either milder infectious insults, preserved immunoregulatory mechanisms, or paradoxically, immunosenescent hyporesponsiveness that, in this context, proves protective.

### Clinical characteristics across pyroptosis phenotypes

The three pyroptosis endotypes exhibited a striking gradient in clinical and biological profiles (**Table 2**). Critically, the Hyper-pyroptotic phenotype (Cluster 2) was not merely an inflammatory extreme; it constituted a syndromic convergence of advanced age, highest disease severity (PSI: 107.81), profound malnutrition (48.2% with MNA-SF ≤7), and a cytokine storm (IL-6 median: 98.4 pg/mL). This phenotype accounted for the majority (75.0%, 42/56) of all 28-day deaths. Conversely, the Hypo-pyroptotic group presented with preserved nutrition and minimal inflammation, experiencing near-universal survival (98.8%). These patterns suggest that the identified endotypes capture integrated host vulnerability, transcending traditional comorbidity-based classifications.

**Table 2:** Clinical characteristics across pyroptosis phenotypes in older adults with CAP.

Variables	Total (n=282)	Cluster 1: iIntermediate-pyroptotic (n=128)	Cluster 2: hyper-pyroptotic (n=73)	Cluster 3: hypo-pyroptotic (n=81)	P
Gender					0.314
Male, n (%)	147 (52.1)	63 (42.9)	36 (24.5)	48 (32.7)	
Female, n (%)	135 (47.9)	65 (48.1)	37 (27.4)	33 (24.4)	
Age, mean (SD)	81.65 (4.41)	81.55 (4.71)	83.47 (4.11)	80.19 (3.56)	<0.001
BMI, kg/m², mean (SD)	22.77 (2.67)	22.37 (2.67)	22.59 (2.76)	23.58 (2.44)	0.005
PSI, mean (SD)	97.98 (14.14)	98.31 (12.62)	107.81 (14.18)	88.61 (9.56)	<0.001
Prognosis					<0.001
Survival, n (%)	226 (80.1)	115 (50.9)	31 (13.7)	80 (35.4)	
28-day mortality, n (%)	56 (19.9)	13 (23.2)	42 (75.0)	1 (1.8)	
Nutritional status					<0.001
Malnutrition MNA-SF ≤7 points, n (%)	106 (37.6)	55 (51.9)	49 (46.2)	2 (1.9)	
At risk of malnutrition MNA-SF 8–11 points, n (%)	99 (35.1)	50 (50.5)	20 (20.2)	29 (29.3)	
Well-nourished MNA-SF12–14 points, n (%)	77 (27.3)	23 (29.9)	4 (5.2)	50 (64.9)	
Underlying diseases					
Hypertension, n (%)	111 (39.4)	46 (41.1)	30 (27.0)	35 (31.5)	0.542
Diabetes, n (%)	102 (36.2)	50 (49.0)	30 (29.4)	22 (21.6)	0.130
Chronic Kidney Disease (CKD Stage 3 and above), n (%)	68 (24.1)	29 (42.6)	21 (30.9)	18 (26.5)	0.557
Cerebrovascular disease, n (%)	74 (26.2)	30 (40.5)	16 (21.6)	28 (37.8)	0.127
COPD, n (%)	99 (35.1)	36 (36.4)	33 (33.3)	30 (30.3)	0.106
Etiology					0.344
Viral pneumonia, n (%)	67 (23.8)	33 (49.3)	14 (20.9)	20 (29.9)	
Bacterial pneumonia, n (%)	66 (23.4)	27 (40.9)	19 (28.8)	20 (30.3)	
Mixed infections, n (%)	50 (17.7)	17 (34.0)	14 (28.0)	19 (38.0)	
Unidentified, n (%)	99 (35.1)	51 (51.5)	26 (26.3)	22 (22.2)	
Laboratory Test Results					
WBC,×10^9^/L, mean (SD)	13.40 (3.55)	13.49 (3.52)	13.52 (4.03)	13.16 (3.16)	0.773
Hemoglobin, g/L, mean (SD)	113.10 (18.46)	113.67 (20.54)	111.86 (17.02)	113.31 (16.28)	0.795
Platelet,×109/L, mean (SD)	173.05 (76.40)	175.78 (78.16)	159.27 (53.35)	181.16 (89.37)	0.178
Albumin, g/L, mean (SD)	32.92 (4.06)	32.90 (4.08)	31.53 (4.01)	34.22 (3.67)	<0.001
IL-6, pg/ml, median (IQR)	56.50 (32.60,113.63)	43.90 (32.10,98.28)	98.40 (55.35,144.41)	42.40 (29.30,77.98)	<0.001
IL-1β, pg/ml, median (IQR)	5.50 (3.56,7.11)	5.99 (4.83,7.23)	6.60 (5.02,7.58)	3.19 (2.44,4.81)	<0.001
IL-18, pg/ml, median (IQR)	426.06 (334.39,523.16)	436.45 (337.20,519.99)	490.83 (409.49,564.80)	373.95 (294.65,468.91)	<0.001

BMI body mass index; PSI Pneumonia Severity Index; MNA-SF Mini Nutritional Assessment–Short Form; CKD chronic kidney disease; WBC white blood cell count; IL-6 interleukin-6; IL-1β interleukin-1β; IL-18 interleukin-18; GSDMD gasdermin D; NLRP3 NOD-like receptor protein 3; CASP1 caspase-1; SD standard deviation; IQR interquartile range.

Categorical variables are presented as n (column %). Continuous variables are summarized as mean ± SD for normally distributed data or median [IQR] for skewed distributions.

Importantly, downstream cytokines not used for clustering—IL-1β (median: 6.60 *vs*. 5.99 *vs*. 3.19 pg/mL), IL-18 (490.83 *vs*. 436.45 *vs*. 373.95 pg/mL), and IL-6 (98.40 *vs*. 43.90 *vs*. 42.40 pg/mL)—exhibited a monotonic gradient across the hyper-, intermediate-, and hypo-pyroptotic endotypes (all P < 0.001), confirming biological concordance between the upstream pyroptosis-defined clusters and their expected downstream inflammatory output.

By contrast, the prevalence of hypertension, diabetes, CKD, cerebrovascular disease, COPD, and the distribution of pneumonia etiology did not differ significantly among clusters. This suggests that the pyroptosis-based phenotypes capture prognostically relevant inflammatory and nutritional differences that are not explained by baseline comorbidities or pathogen type.

### Survival outcomes across pyroptosis phenotypes

**Figure 3:**
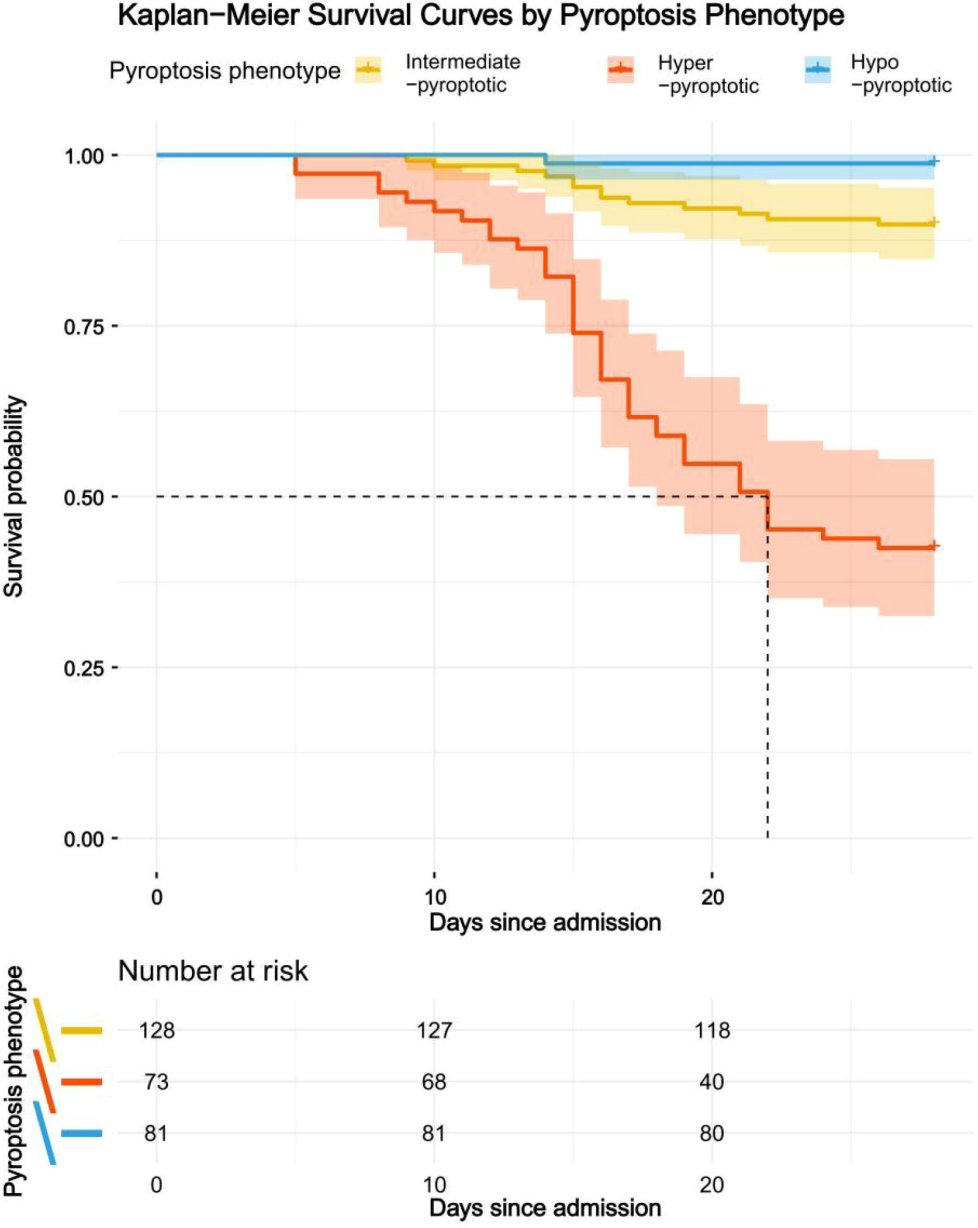
Kaplan-Meier survival curves for the three pyroptosis phenotypes. Patients were stratified based on their pyroptosis-related protein expression clusters. The Hyper-pyroptotic phenotype (red) was associated with the poorest survival, followed by the Intermediate-pyroptotic (gold) and Hypo-pyroptotic (blue) phenotypes. The difference in survival distributions was highly significant (P < 0.001, log-rank test). Tick marks on the curves represent censored observations.

Kaplan-Meier survival analysis demonstrated significant disparities in post-admission survival among the three pyroptosis phenotypes (P < 0.001 by the log-rank test) (**Figure 3**). Patients in the Hyper-pyroptotic group experienced the most unfavorable prognosis, with a median survival time of 21.03 ± 0.82 days and only 42.5% survival probability at 28 days. In contrast, the Hypo-pyroptotic group exhibited the most favorable outcomes, with over 98.8% of patients surviving during the follow-up period. The Intermediate-pyroptotic group showed a survival trajectory between the other two groups, with 89.8% of patients surviving during the follow-up period. Pairwise comparisons confirmed that survival differences between each pair of clusters were statistically significant (all P < 0.01).

### Correlations between pyroptosis markers, inflammation, nutrition, and disease severity

**Figure 4:**
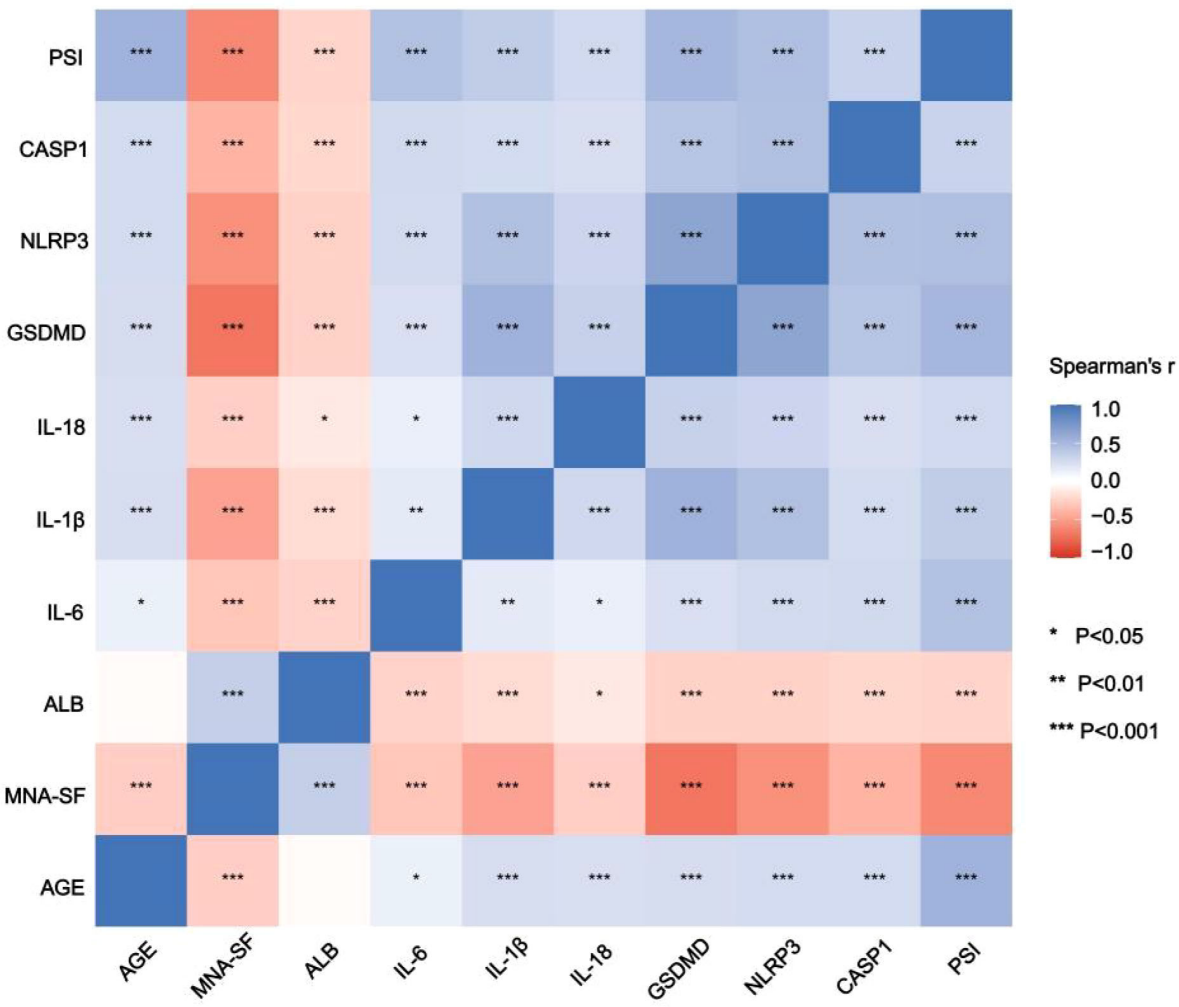
Correlation heatmap of pyroptosis, inflammatory markers, nutritional status and disease severity. Abbreviations: PSI Pneumonia Severity Index; MNA-SF Mini Nutritional Assessment–Short Form; IL-6 interleukin-6; IL-1β interleukin-1β; IL-18 interleukin-18; GSDMD gasdermin D; NLRP3 NOD-like receptor protein 3; CASP1 caspase-1. *P,0.05, **P,0.01, ***P,0.001.

The results of the Spearman correlation analysis indicated that various significant correlations were observed among different indicators (**Figure 4**). Among pyroptosis-related factors, GSDMD, NLRP3, and CASP1 exhibited a strong positive correlation with each other (all P<0.001), suggesting a tendency for synergistic activation of pyroptosis signaling molecules in older adults with CAP. In addition, CASP1 showed significant positive correlations with inflammatory cytokines IL-1β (r=0.551, P<0.001), IL-18 (r=0.337, P<0.001), and IL-6 (r=0.237, P<0.001), indicating a close association between the pyroptosis pathway and the inflammatory response. Regarding nutritional status and disease severity indicators, MNA-SF was significantly correlated with ALB (r=0.345, P<0.001) as well as GSDMD (r=-0.703, P<0.001). The PSI showed moderate positive correlations with several inflammation or pyroptosis markers (such as CASP1, NLRP3, and GSDMD) (all P<0.01), further underscoring the important impact of pyroptosis and inflammation activation on disease progression and outcomes in patients.

### Nonlinear and joint prognostic effects of nutrition and pyroptosis

**Figure 5:**
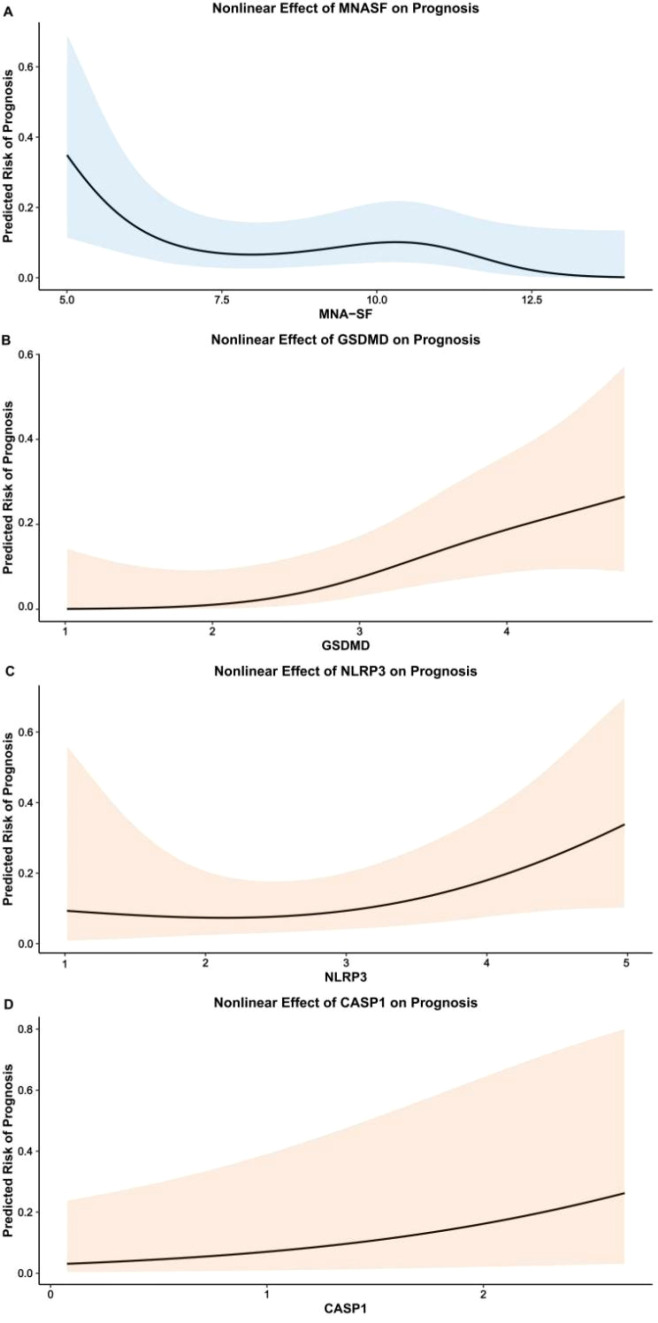
Nonlinear partial effects of nutrition and pyroptosis biomarkers on 28-day mortality in older adults with CAP. **(A)** s(MNA-SF, k=4), edf = 2.67, P = 0.038; **(B)** s(GSDMD, k=4), edf = 1.81, P = 0.020; **(C)** s(NLRP3, k=4), edf = 1.66, P = 0.048; **(D)** s(CASP1, k=4), edf = 1.00, P = 0.015. Curvature in panels A–C indicates departures from linearity. Panel D approximates a linear effect. Each panel shows the estimated smooth on the logit scale (y-axis; partial effect centered at 0) against the predictor value (x-axis; standardized to z-scores). Solid lines denote the point estimate; shaded bands indicate 95% confidence intervals; rug marks indicate observed data density. Binomial family with logit link; thin-plate regression splines with maximum basis dimension k=4; smoothness selected by REML; adjusted for age and sex. Deviance explained = 39.4%; adjusted R² = 0.401; n = 282.

In the primary GAM, age (P = 0.764) and sex (P = 0.908) did not show significant linear associations with 28-day mortality. By contrast, in **Figure 5**, the smooth terms for MNA-SF (edf=2.67, χ²=7.45, P = 0.038), GSDMD (edf=1.81, χ²=8.38, P = 0.020) and NLRP3 (edf=1.66, χ²=6.19, P = 0.048) were statistically significant, indicating nonlinear effects, whereas CASP1 displayed a significant, approximately linear association (edf=1.00, P = 0.015). The model explained 39.4% of deviance (adjusted R²=0.401; n=282).

The partial effect curves revealed clinically interpretable nonlinear patterns (**Figure 5**). For MNA-SF, mortality risk remained relatively stable across the normal nutritional range (scores 12–14) but escalated sharply below a threshold of approximately 8 points, with each additional point decrease associated with progressively larger increments in log-odds of death—a pattern consistent with exhaustion of nutritional reserve.

The partial effect curves for GSDMD and NLRP3 revealed a clinically significant pattern: mortality risk increased steeply through the low-to-moderate concentration range but exhibited attenuation at higher values (**Figures 5B, C**). This saturation phenomenon has important implications for both prognostication and therapeutic targeting. From a prognostic standpoint, once GSDMD exceeds approximately 3.5 ng/mL (standardized z-score ~1.5), further elevations provide diminishing incremental information about mortality risk—patients above this threshold are already at substantially elevated risk regardless of exact biomarker values. This threshold pattern may be useful for risk stratification, particularly among patients meeting established MNA-SF criteria for malnutrition. Likewise, the observed saturation pattern suggests that the prognostic information carried by these biomarkers is concentrated within specific ranges; whether these ranges correspond to modifiable biological states requires prospective study.

**Figure 6:**
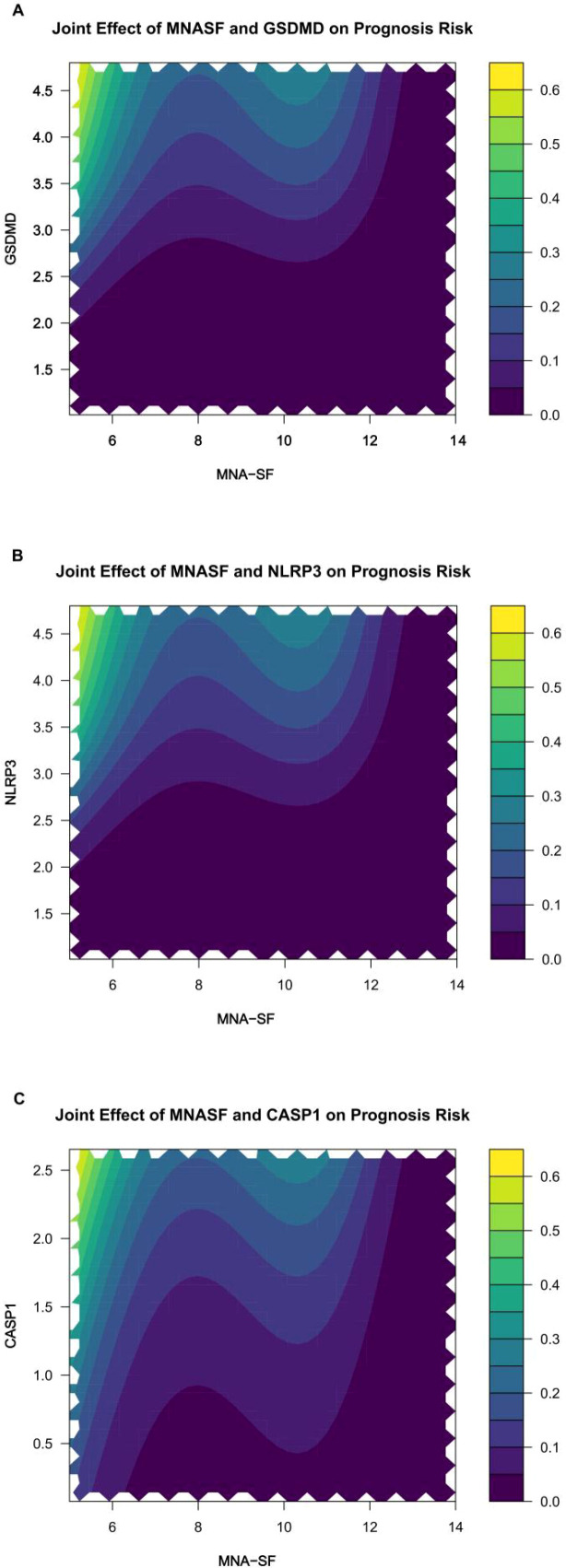
Exploratory two−dimensional partial dependence surfaces for joint patterns between nutrition and pyroptosis biomarkers. **(A)** MNA−SF × GSDMD; **(B)** MNA−SF × NLRP3; **(C)** MNA−SF × CASP1. Colored surfaces (warmer colors = higher log−odds of 28−day mortality) with overlaid contour lines. Axes are standardized (z−scores). Other covariates are held at their median (continuous) or reference level (categorical).

Two-dimensional partial dependence surfaces (**Figure 6**) revealed potentially synergistic risk patterns: patients occupying the low MNA-SF/high GSDMD quadrant demonstrated predicted mortality probabilities substantially exceeding what would be expected from additive combination of individual risk contributions. This observation, while exploratory and requiring validation, suggests that nutritional compromise may amplify the deleterious effects of pyroptosis dysregulation—a hypothesis consistent with the “multiple-hit” paradigm in geriatric pathophysiology.

### LASSO−selected prognostic model with high discrimination and calibration in older adults with CAP

**Figure 7:**
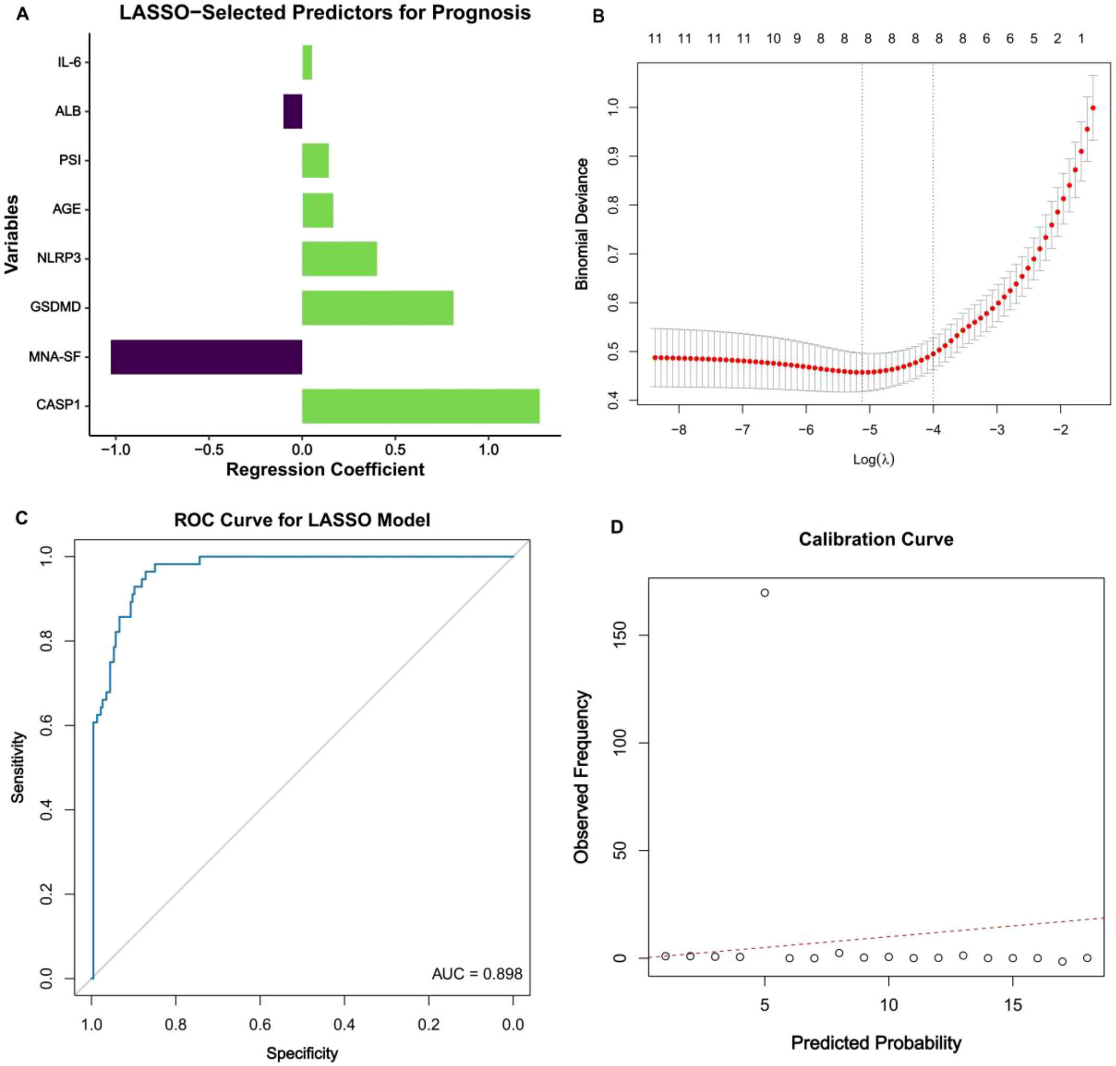
LASSO-selected prognostic model for 28-day mortality in older adults with CAP. Panel **(A)** (Non-zero coefficients at λ.1se): Bar plot of standardized LASSO coefficients for predictors retained at the prespecified λ.1se penalty. Positive bars indicate higher log-odds of 28-day mortality; negative bars indicate lower log-odds. Domains are color-coded: demographic (age), clinical severity (PSI), nutritional (MNA-SF, albumin), inflammatory/pyroptosis (IL-6, GSDMD, NLRP3, CASP1). Panel **(B)** (10-fold cross-validation curve): Mean binomial deviance (points with 1-SE bands) plotted against log(λ). Vertical dotted lines mark λ.min (minimum mean deviance) and λ.1se (one-standard-error rule used for model selection). Panel **(C)** (Discrimination): ROC curve for the refit multivariable logistic model; the solid curve shows sensitivity versus 1-specificity across probability thresholds, with AUC = 0.898. The diagonal line represents no-discrimination. Panel **(D)** (Calibration): Calibration plot comparing predicted versus observed 28-day mortality. Points represent deciles of predicted risk; the solid curve is a loess fit with 95% confidence band. The dashed 45° line denotes perfect calibration. Hosmer–Lemeshow test: X² = 5.707, df = 8, P = 0.680. Interpretation: Predictions closely track observed outcomes across risk strata.

We constructed a prognostic model for 28−day mortality using penalized logistic regression with least absolute shrinkage and selection operator (LASSO). Consistent with the prespecified analysis plan, all continuous predictors were standardized to z−scores before modeling, and the penalty parameter was chosen by the λ.1se criterion from 10−fold cross−validation. At this penalty, eight predictors had non−zero coefficients: age, MNA−SF, PSI, albumin, IL−6, GSDMD, NLRP3, and CASP1 (**Figures 7A, B**). These variables were then refit in a multivariable logistic regression to obtain final coefficient estimates and odds ratios for interpretation.

The resulting model achieved high discrimination, with an area under the receiver operating characteristic curve (AUC) of 0.898 (**Figure 7C**), indicating strong separation between survivors and non−survivors at 28 days. Calibration analysis demonstrated close agreement between predicted and observed risks across the spectrum of predicted probabilities (**Figure 7D**). The Hosmer–Lemeshow goodness−of−fit statistic was X² = 5.707 with 8 degrees of freedom (P = 0.680), providing no evidence of lack of fit. Together, these results indicate that a compact set of demographic, clinical severity, nutritional, inflammatory, and pyroptosis−related variables yields a well−calibrated model with high discriminatory capacity for short−term prognosis in older adults hospitalized with CAP.

### Predictive performance and clinical utility

The LASSO-selected prognostic model incorporating age, nutritional status (MNA-SF), disease severity (PSI), albumin, IL-6, and pyroptosis-related biomarkers (GSDMD, NLRP3, CASP1) demonstrated excellent discriminatory performance for 28-day mortality, with an area under the receiver operating characteristic curve (AUC) of 0.898 (95% CI: 0.847–0.943). This significantly outperformed the Pneumonia Severity Index (PSI) alone, which achieved an AUC of 0.793 (95% CI: 0.744–0.841; P < 0.001 for difference).

**Figure 8:**
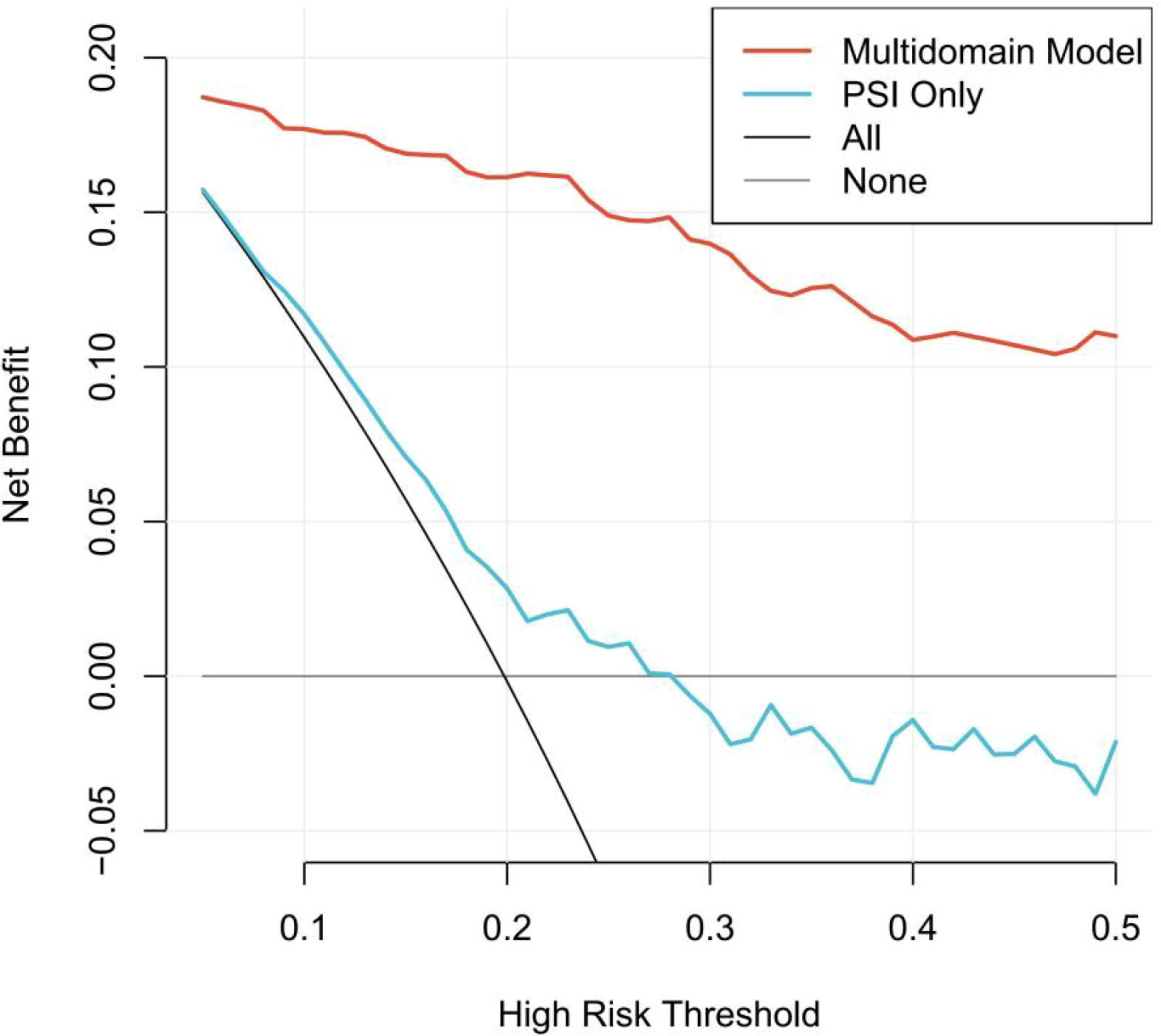
DCA comparing clinical utility of prediction models for 28-day mortality. The multidomain model (red line) demonstrates superior net benefit across clinically relevant threshold probabilities compared to the PSI alone (blue line) and default strategies of treating all patients (grey dashed line) or treating no patients (black line).

Decision curve analysis revealed superior clinical utility of the LASSO-selected prognostic model across a clinically relevant range of threshold probabilities (10%–30%) (**Figure 8**). At a 10% decision threshold—where clinicians might consider intensified monitoring—the multidomain model provided a net benefit of 0.177, compared to 0.115 for PSI alone (net benefit difference: 0.061). The advantage became more pronounced at higher thresholds: at 20% (threshold for considering escalation of care), the net benefit was 0.161 for the multidomain model versus 0.025 for PSI (difference: 0.137); at 30% (threshold for considering ICU admission or aggressive interventions), the multidomain model maintained a positive net benefit of 0.140, while PSI yielded a negative net benefit of -0.016 (difference: 0.156).

Throughout the threshold range where clinical decisions are most uncertain (10%–30%), the multidomain model consistently provided higher net benefit than both the treat-all and treat-none strategies, as well as the PSI-based approach. This suggests that integrating nutritional and pyroptosis biomarkers with conventional clinical parameters not only improves statistical prediction accuracy but also enhances practical clinical decision-making for risk stratification in older adults with CAP.

## Discussion

This study provides three principal findings with implications for understanding and predicting outcomes in geriatric pneumonia. First, we demonstrate that older adults with CAP harbor biologically distinct pyroptosis endotypes—not a continuous spectrum—with profoundly divergent mortality trajectories ranging from 1.2% in the hypo-pyroptotic phenotype to 57.5% in the hyper-pyroptotic phenotype. Second, the relationships between pyroptosis biomarkers and mortality follow nonlinear patterns characterized by threshold-dependent risk escalation and saturation effects—patterns obscured by conventional linear modeling approaches. Third, we observed that nutritional status modified the association between pyroptosis biomarkers and 28-day mortality, with the highest predicted risks occurring when malnutrition and elevated pyroptosis markers co-occurred. These findings are hypothesis-generating and do not establish causality, but they support a framework in which nutritional vulnerability and inflammasome-related injury may be biologically linked.

### Pyroptosis biomarkers: nonlinear risk and context within prior work

Our results add human, geriatric evidence to a literature that has been driven largely by experimental models. In pneumonia, NLRP3 inflammasome activation can be beneficial for host defense yet harmful when excessive, worsening lung injury (11, 34, 35). The nonlinear biomarker–mortality patterns observed here offer a unifying interpretation: modest elevations may reflect appropriately engaged innate immunity, whereas higher levels are more consistent with dysregulated, tissue-injurious inflammation. Such threshold-type behavior is easy to miss with linear assumptions or dichotomized cut-points, which may partly explain the inconsistent prognostic signals reported previously.

The link between poorer nutritional status and higher pyroptosis markers also suggests that these processes are biologically coupled rather than parallel, independent risk pathways. Malnutrition may weaken autophagy-dependent restraint of inflammasome activity and, through micronutrient deficits, lower the activation threshold of NLRP3 signaling. In older adults, age-related immune remodeling may further prime inflammasome pathways (36, 37), providing a plausible backdrop for the steep risk gradients concentrated within specific biomarker ranges.

Consistent with this biology, generalized additive models revealed sharp increases in mortality risk across intermediate ranges of GSDMD and NLRP3, followed by a plateau at extreme values—patterns that conventional linear models would obscure (38). This saturation is plausible if downstream pyroptotic signaling becomes functionally maximal beyond a certain point, limiting the incremental prognostic value of further biomarker increases (39, 40). Our findings also align with reports linking elevated GSDMD to worse outcomes in other acute inflammatory states such as COVID-19 and sepsis (41, 42), while extending prior work by jointly profiling multiple nodes of the pyroptosis cascade and explicitly modeling nonlinearity to better reflect inflammasome dynamics in clinical disease.

Prior studies in pneumonia, sepsis, and ARDS have demonstrated that plasma levels of pyroptosis-related proteins—including GSDMD, caspase-1, and IL-18—correlate with bronchoalveolar lavage fluid concentrations and pulmonary injury severity. Specifically, serum GSDMD levels have been shown to positively correlate with chest CT consolidation area in COVID-19 pneumonia (r = 0.56, P < 0.001) (43), and BALF concentrations of NLRP3 significantly exceed paired serum levels in ARDS patients, confirming the lung as a primary site of inflammasome activation (44). In a VILI mouse model, mechanical ventilation simultaneously elevated IL-18 in lung tissue, serum, and BALF, establishing that pulmonary inflammasome activation directly contributes to circulating cytokine levels (45). Furthermore, active GSDMD has been detected in plasma exosomes derived from activated monocytes in septic patients, many with pulmonary infection sources, and elevated serum GSDMD predicts ICU admission, mechanical ventilation requirement, and mortality in pneumonia patients (46). These data collectively support—but do not prove—that circulating biomarkers serve as accessible surrogates of compartmentalized lung inflammasome activity.

### Nutritional status: independent association and conceptual link to cell-death pathways

Malnutrition, quantified by MNA-SF and corroborated by serum albumin, independently predicted 28-day mortality after multivariable adjustment, in line with literature demonstrating that undernutrition impairs host defense, exacerbates sarcopenia, and delays recovery from respiratory infections in older adults (47, 48). The interplay between malnutrition and dysregulated inflammation is particularly relevant in geriatric populations. In our data, low MNA−SF scores correlated with lower albumin, higher GSDMD levels and higher PSI scores, and exploratory two−dimensional partial−dependence plots suggested that patients with both severe nutritional compromise and elevated pyroptosis markers bear the highest short−term risk. This pattern aligns with the “multiple−hit” paradigm in geriatric immunology, in which diminished nutritional reserves, sarcopenia and chronic low−grade inflammation (“inflammageing”) jointly reduce resilience to acute stressors (49–51). Clinically, these findings support early nutritional risk screening in older adults hospitalized with CAP. They also motivate prospective studies to test whether optimizing nutrition, alone or alongside host-directed modulation of inflammasome/IL-1 signaling, can improve outcomes in biomarker-defined high-risk subgroups (52).

### Mechanistic convergence of malnutrition and pyroptosis dysregulation

The association between nutritional compromise and heightened pyroptosis activity suggests convergence at several immunoregulatory pathways. One plausible mechanism involves impaired autophagy. Protein–energy malnutrition suppresses autophagy-related gene expression and disrupts autophagic flux, limiting the clearance of damaged mitochondria and protein aggregates (53, 54). Notably, autophagy declines progressively with aging, and this age-related impairment is further exacerbated by nutritional deficiency, creating a compounding vulnerability in malnourished older adults. Experimental evidence indicates that restoring autophagy competence can attenuate age-associated inflammasome hyperactivation and extend lifespan (55), underscoring the functional relevance of this pathway.

Accumulation of dysfunctional mitochondria promotes the release of reactive oxygen species and mitochondrial DNA, both of which are potent activators of NLRP3 inflammasome assembly (23, 56).

Micronutrient deficiencies may further lower the threshold for inflammasome activation. Zinc deficiency—common in malnourished older adults—disrupts key negative regulators of NLRP3 signaling, including A20, thereby facilitating inflammasome oligomerization (25, 57). In parallel, vitamin D insufficiency, prevalent in hospitalized elderly populations, removes constitutive inhibitory control over NLRP3 transcription and priming (58, 59).

A third, complementary pathway involves disruption of the gut–lung axis. Malnutrition-associated intestinal dysbiosis and increased gut permeability enhance systemic exposure to lipopolysaccharide, potentially priming alveolar macrophages toward exaggerated pyroptotic responses upon pulmonary infection (60, 61).

**Figure 9:**
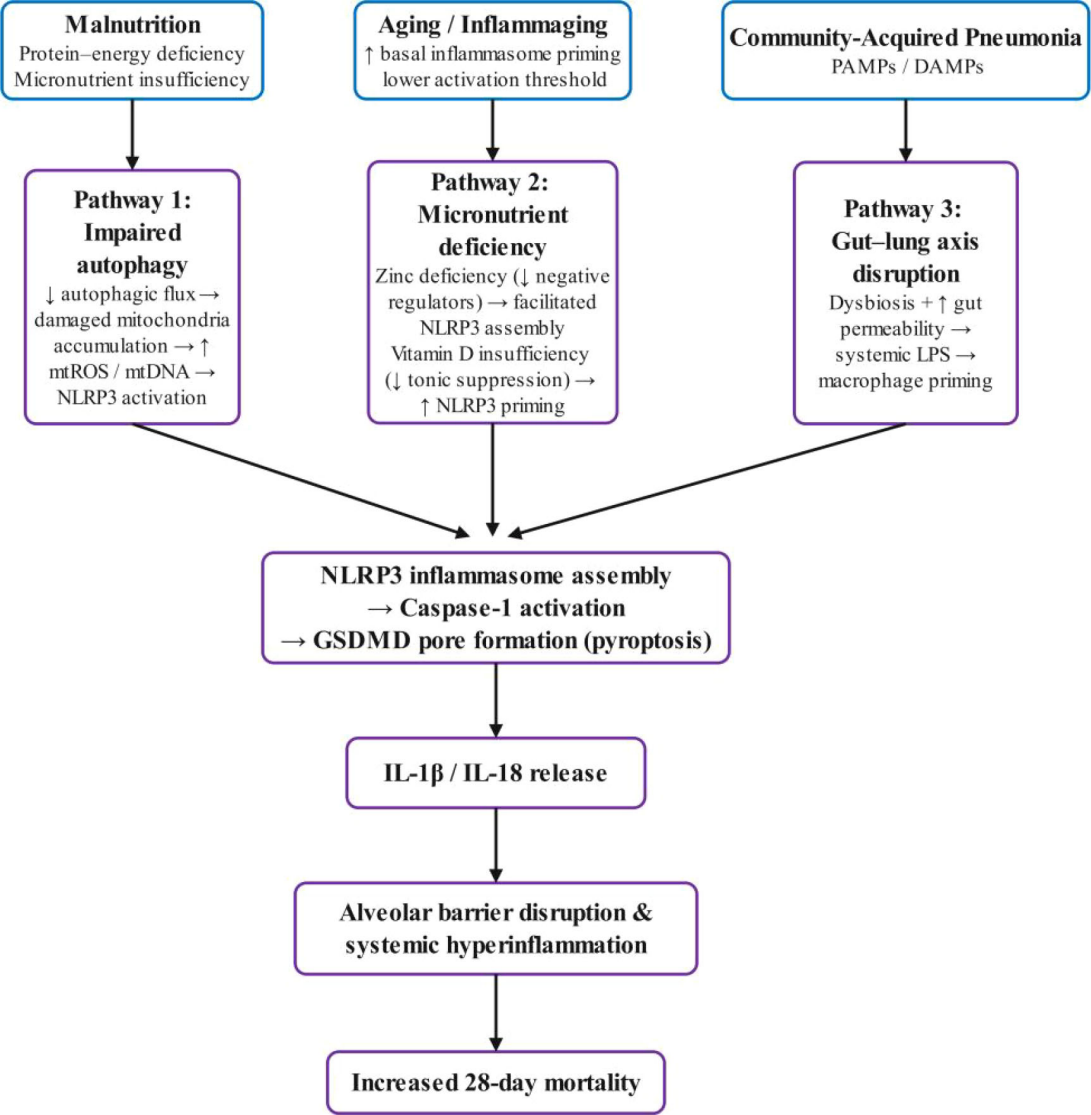
Proposed pathophysiological model: mechanistic convergence of malnutrition and pyroptosis dysregulation in geriatric community-acquired pneumonia.

Consistent with this framework, our two-dimensional analyses revealed supra-additive mortality risk among patients with concurrent malnutrition and elevated GSDMD levels, supporting a “priming–triggering” model of pyroptosis dysregulation. Although causality cannot be inferred from the cross-sectional design, these findings provide biologically coherent and testable hypotheses for future mechanistic and interventional studies. The proposed pathophysiological model integrating these three mechanistic pathways—impaired autophagy, micronutrient deficiency, and gut–lung axis disruption—is schematically summarized in **Figure 9**.

### Clinical implications

Risk Identification. At a 20% mortality threshold—a clinically relevant trigger for care escalation—the multidomain model yielded a net benefit of 0.137 compared with PSI alone. In practical terms, across 1,000 hypothetical patients, this corresponds to identifying about 137 additional high-risk individuals for closer attention without increasing false-positive classifications.

Care Pathway Design. The presence of distinct pyroptosis endotypes suggests that older adults hospitalized with CAP represent biologically heterogeneous subgroups. If confirmed in external cohorts, these endotypes may support risk-informed decisions about monitoring intensity and early multidisciplinary assessment (including nutrition), rather than implying endotype-specific treatment strategies or discharge pathways based on our observational data.

Therapeutic Hypothesis Generation. The nonlinear NLRP3–mortality pattern, including apparent risk saturation at higher concentrations, may help define testable “windows” for biomarker-guided, host-directed interventions. However, whether nutritional optimization or inflammasome/IL-1 pathway–targeted therapies improve outcomes—and in which biomarker ranges—requires prospective, randomized evaluation.

### Study limitations

Several limitations merit consideration. First, the study lacks external validation. Although the LASSO model demonstrated good internal discrimination and calibration, these estimates are prone to optimism when evaluated in the derivation cohort alone. Notably, with 56 events and 8 retained predictors, the events-per-variable ratio (~7) is acceptable for penalized methods but falls below the conventional threshold of 10 for unpenalized logistic regression. We did not split the dataset given the modest sample size; however, internal validation—however rigorous—cannot substitute for prospective external validation, which is required before clinical application.

Second, residual confounding cannot be excluded. We adjusted for a broad set of covariates, but no validated frailty instrument (e.g., Clinical Frailty Scale, Fried phenotype, or Katz ADL) was routinely administered during the study period, nor could such data be reliably extracted retrospectively. This is relevant because frailty shares upstream drivers with malnutrition—including sarcopenia and chronic inflammation—so MNA-SF scores may partly capture frailty-related vulnerability rather than nutritional deficit alone. Furthermore, frailty-associated low-grade inflammation could amplify baseline inflammasome priming, thereby shaping the pyroptosis profiles that underlie our clusters (62). Several covariates in our models (age, BMI, PSI, albumin, comorbidity burden) overlap with frailty domains and likely attenuate this confounding, yet they cannot replace formal frailty assessment. The independent prognostic contributions attributed to malnutrition and pyroptosis markers may therefore be overestimated. Prospective studies embedding standardized frailty screening at enrollment are needed to disentangle the respective roles of frailty, nutritional status, and inflammasome activation.

Third, pyroptosis biomarkers were measured at a single early time point. Because pre-admission symptom duration was not reliably documented, patients may have been sampled at different illness phases, and circulating inflammasome-related proteins evolve over the course of acute infection. Although 78.4% of patients were sampled before antibiotic initiation, the remaining 21.6% had already received empirical therapy, which may attenuate pathogen-driven inflammasome stimulation within hours. These concerns are partially offset by the standardized admission sampling protocol, the large biomarker separations across endotypes that far exceed the variability expected from modest timing differences within a 24-hour window, and high bootstrap stability of cluster assignments (mean ARI = 0.78). Nonetheless, prospective studies should record the timing of symptom onset, blood sampling, and antibiotic initiation, and ideally incorporate serial sampling to track inflammasome activation longitudinally.

Fourth, serum albumin is difficult to interpret in acute infection because it reflects both nutritional status and negative acute-phase responses. While we paired albumin with MNA-SF to capture complementary nutritional dimensions, the cross-sectional design cannot fully separate inflammation-driven changes from pre-existing malnutrition.

Fifth, as a single-center study in a Chinese tertiary hospital with a predominantly Han Chinese cohort, generalizability may be limited. Ethnic differences in inflammasome-related polymorphisms (e.g., NLRP3, CASP1) may shift baseline activation thresholds (63), and population variation in micronutrient status—particularly zinc and vitamin D—may influence both malnutrition prevalence and the magnitude of malnutrition–pyroptosis interactions (64). The tertiary setting likely enriched for higher-severity illness, and differences in hospitalization thresholds across health systems may alter biomarker distributions and endotype prevalence. In addition, incomplete microbiological confirmation—common in older adults with CAP (58, 59)—prevented pathogen-specific analyses and leaves open the possibility of etiology-dependent pyroptosis dynamics. External validation in ethnically and geographically diverse cohorts is therefore needed before applying the proposed biomarker thresholds broadly.

Sixth, circulating pyroptosis markers may not exclusively reflect pulmonary inflammasome activity, as systemic sources (e.g., circulating monocytes, hepatic or splenic macrophages) likely contribute to plasma levels. Direct assessment of compartmentalized pulmonary pyroptosis through bronchoalveolar lavage or lung tissue analysis was not feasible in this retrospective study.

Finally, the three-cluster endotype solution should be viewed as hypothesis-generating. Although bootstrap and sensitivity analyses supported stability, unsupervised clustering is inherently data-dependent, and alternative structures—including two- or four-cluster solutions—may emerge in different populations. Establishing the biological relevance of these endotypes will require mechanistic studies in relevant cells and tissues.

Despite these constraints, our findings support the prognostic value of integrating nutritional status with pyroptosis biology in geriatric CAP and provide a foundation for targeted validation and mechanistic work.

### Future perspectives

Future work should focus on multicenter, prospective studies to validate these endotypes and prognostic models in broader, more heterogeneous cohorts of older adults with CAP. External validation should include not only discrimination and calibration, but also whether decision-curve gains translate into meaningful changes in clinical decisions and patient outcomes. Studies with standardized documentation of symptom onset, antibiotic timing, and phlebotomy time—ideally with serial sampling over the first days of hospitalization—are needed to characterize biomarker trajectories and clarify when pyroptosis-related signals are most informative. Finally, our findings motivate biomarker-informed interventional trials to test whether protocolized nutritional optimization and/or host-directed modulation of inflammasome/IL-1 signaling improves outcomes in prespecified high-risk subgroups. Integrating proteomic or metabolomic profiling may further refine risk stratification and help link clinical phenotypes to underlying biology through multidisciplinary collaboration.

## Conclusion

In older adults with community-acquired pneumonia, pyroptosis biomarkers define distinct endotypes with markedly divergent mortality trajectories. Biomarker-outcome relationships follow nonlinear patterns, and malnutrition amplifies pyroptosis-associated risk through apparent synergistic interactions. Integration of nutritional assessment with pyroptosis biomarkers significantly enhances mortality prediction beyond conventional clinical scores. While these findings await external validation, they support a multidimensional approach to risk stratification that reflects key pathobiological pathways in geriatric pneumonia.

## Data Availability

The raw data supporting the conclusions of this article will be made available by the authors, without undue reservation.
